# Acute and Chronic Sleep Deprivation-Related Changes in N-methyl-D-aspartate Receptor—Nitric Oxide Signalling in the Rat Cerebral Cortex with Reference to Aging and Brain Lateralization

**DOI:** 10.3390/ijms20133273

**Published:** 2019-07-03

**Authors:** Zdenka Kristofikova, Jana Sirova, Jan Klaschka, Saak V. Ovsepian

**Affiliations:** 1 National Institute of Mental Health, Topolova 748, 250 67 Klecany, Czech Republic; 2 Institute of Computer Science, Academy of Sciences of the Czech Republic, Pod vodarenskou vezi 2, 182 07 Prague 8, Czech Republic; 3 Department of Psychiatry and Medical Psychology, 3rd Faculty of Medicine of Charles University, 11636 Prague, Czech Republic; 4 International Centre for Neurotherapeutics, Dublin City University, 9 Dublin, Ireland

**Keywords:** aging, acute and chronic sleep deprivation, cortex, brain lateralization, NMDA receptor subunits, nitric oxide synthases

## Abstract

Aging and chronic sleep deprivation (SD) are well-recognized risk factors for Alzheimer’s disease (AD), with N-methyl-D-aspartate receptor (NMDA) and downstream nitric oxide (NO) signalling implicated in the process. Herein, we investigate the impact of the age- and acute or chronic SD-dependent changes on the expression of NMDA receptor subunits (NR1, NR2A, and NR2B) and on the activities of NO synthase (NOS) isoforms in the cortex of Wistar rats, with reference to cerebral lateralization. In young adult controls, somewhat lateralized seasonal variations in neuronal and endothelial NOS have been observed. In aged rats, overall decreases in NR1, NR2A, and NR2B expression and reduction in neuronal and endothelial NOS activities were found. The age-dependent changes in NR1 and NR2B significantly correlated with neuronal NOS in both hemispheres. Changes evoked by chronic SD (dysfunction of endothelial NOS and the increasing role of NR2A) differed from those evoked by acute SD (increase in inducible NOS in the right side). Collectively, these results demonstrate age-dependent regulation of the level of NMDA receptor subunits and downstream NOS isoforms throughout the rat brain, which could be partly mimicked by SD. As described herein, age and SD alterations in the prevalence of NMDA receptors and NOS could contribute towards cognitive decline in the elderly, as well as in the pathobiology of AD and the neurodegenerative process.

## 1. Introduction

Sporadic Alzheimer’s disease (AD) is the most common form of dementia, with its causes remaining to be established. Interactions among multiple genetic, epigenetic, and environmental factors seem to play a role, especially in late-onset forms. Since progression of AD is related to age, great attention is now focussed on the research of various age-related contributors, which could be controlled and perhaps managed. Among these, healthy aging and lifestyle, as well as optimal sleep, are of major relevance.

Chronic sleep deprivation (SD) appears to be one of the potential environmental risk factors for AD, with sleep disruptions and fragmentations reported from an early stage. Clinical data indicate that about 45% of AD patients have sleep impairments [1]. Alterations in the sleep–wake cycle were also found in transgenic animal models of AD [2,3]. Disruption of sleep is known to interfere with learning and memory, among others, through oxidative stress [4], with the latter known to play a key role in the age-related neurodegenerative process as well, with amyloid β peptide (Aβ) acting as a key inducer of oxidative damage [5,6]. Recently, correlation between the physiological levels of Aβ and the sleep–wake cycle have been also suggested [1], with acute SD significantly increasing total Aβ levels, while chronic SD promotes Aβ aggregation in animals [7]. Chronic SD may also enhance Aβ deposition in human brain [8]. Sleep hormones such as orexin and melatonin (both involved in sleep regulation) applied in vivo in various transgenic animal models of AD or in vitro on hippocampal neurons can influence levels of soluble Aβ, or alleviate its disruptive effects. In the context of plaque formation, their therapeutic use has been considered as an effective inhibitor of nucleation of plaques, but ineffective after plaque formation [7,9,10,11,12,13,14]. Finally, the link between SD and protein tau has been shown recently, with chronic SD of adult or old transgenic animals increasing the level of phosphorylated tau [15,16]. Supplementation of melatonin in vivo has been also reported to improve working memory and arrest hyperphosphorylation of tau [17,18].

The N-methyl-D-aspartate (NMDA) receptor–nitric oxide (NO) pathway is involved in both the sleep–wake cycle and AD pathobiology. Glutamate is the most common excitatory neurotransmitter in the brain, activating NMDA (and several other) receptors [19]. Surprisingly, little is known about glutamatergic regulation of sleep and wakefulness. It has been reported that levels of glutamate in some areas of the rat cortex increase during wakefulness and rapid eye movement (REM) sleep, and decrease during non-REM sleep [20], with SD over 6–12 h increasing glutamate levels in the hippocampus and thalamus [21]. During 3 h SD period, glutamate levels in the prefrontal cortex initially exhibit the progressive rise, however, as SD increases, the levels cease to increase and start to decrease [22]. It is suggested that SD can influence NMDA receptors, especially via alterations in surface expression or subunits composition, particularly of NR1, NR2A, and NR2B subunits [23]. In the rat hippocampus, 4 h SD increases NR2A/NR2B ratio as well as total NR2A, whereas 5 h SD does not induce changes. Prolonged (72 h) SD, on the other hand, reduces surface expression of NR1 and NR2A subunits of NMDA receptors. NR2B expression is also decreased in the hippocampus because of SD [23,24,25]. In the rat cortex, 8 h SD leads to upregulation of NR2A subunit transcripts [26].

Similar to sleep, subunits composition of NMDA receptors is altered during normal aging and AD. In the cortex or hippocampus of old compared to young rodents, NR1 levels are reduced, while NR2A or NR2B levels do not seem to alter consistently [27,28,29]. Indeed, NR2B levels were reduced, e.g., in the cortex, but increased in the hippocampus [27]. In autoptic human hippocampi with AD, both mRNA or protein expressions of NR1, NR2A, and NR2B subunits were lower than in age-related controls, although there have been conflicting data here as well [30,31,32]. It is suggested that AD-evoked changes in NMDA receptors could result from direct effects of oligomeric Aβ. Experiments on cell cultures indicate that Aβ oligomers can activate NMDA receptors through both NR1/NR2A or NR1/NR2B heteromers [33,34], with downstream changes in signalling pathways [35]. With respect to brain lateralization, our earlier analysis did not reveal significant differences between the right (R) and left (L) frontal cortices of young male Sprague-Dawley rats in NR1, NR2A, or NR2B subunit expressions [29]. Only mild L/R laterality (index of laterality < +0.100) was found in NR2B expression in the frontal cortex of young male Long Evans rats [36]. On the other hand, there seems to be the same asymmetry in NR2B expression in the hippocampus, reported at a synaptic level [37].

Age- and SD-dependent changes in NMDA receptors raise an intriguing possibility of alteration in NOS signalling, a key molecular partner of NMDA receptor and a major regulator of neural activity [29,38], including adjustments of the sleep–wake cycle, via inducible NOS (iNOS). It is suggested that NO elevation via iNOS is a specific homeostatic mechanism for producing non-REM recovery of sleep following SD [39,40,41]. Neuronal NOS (nNOS) seems to play a complementary role in the recovery of sleep induction processes [39,42]. Although the physiological role of endothelial NOS (eNOS) in the sleep–wake cycle is not known yet in a detail, this isoform appears to be very vulnerable to effects of SD, with 24 h general SD reducing endothelial-dependent vasodilation [43]. Moreover, normal aging impairs the mechanism by which NO induces sleep [44]. Previously, we have compared age-related changes in activities of nNOS, eNOS, and iNOS in Sprague-Dawley, Wistar, and Long Evans rats and found the most marked bilateral changes in the activity of eNOS when compared to nNOS or iNOS [29,38]. On the other hand, analysis of human autoptic hippocampus revealed a lateral asymmetry in eNOS or iNOS activities in nondemented controls and increased activities of nNOS, eNOS, and iNOS, especially in the L hemisphere in subjects with AD, in line with the notion of higher susceptibility of L hemisphere to aging and AD [45]. Thus, our previous results suggested that SD-evoked effects could be different in the R and L hemisphere in the case of NOS isoforms, rather than in NMDA receptor subunits.

This study extends the analysis of NR1, NR2A, and NR2B expressions with nNOS, eNOS, and iNOS activities in the cortex of young and old rats exposed to acute or chronic SD, to elucidate their changes related with aging and sleep impairments, with implications for age-related cognitive decline and pathobiology of AD.

## 2. Results

### 2.1. Changes in NMDA Receptors Subunits and NOS Activities Related to FL (Experiment I)

**Figure 1:**
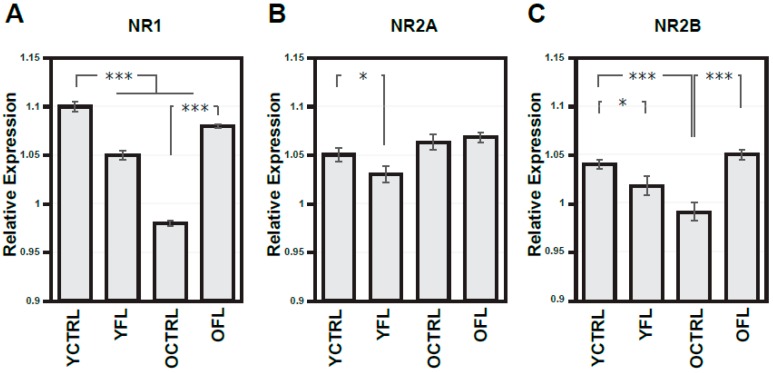
Expressions of N-methyl-D-aspartate receptor (NMDA) receptor subunits in the frontal cortex from both hemispheres in young adult and old rats exposed to forced locomotion. Optical density of respective NMDA subunit bands (**A**–**C**) related to that of α-tubulin. Results are presented as means ± SEM. Statistical significance (Student’s *t*-test) was calculated with respect to young adult or old controls (* *p* < 0.05, *** *p* < 0.001). FL—forced locomotion, YCTRL—young adult controls (*n* = 8), YFL—young adult rats exposed to FL (*n* = 10), OCTRL—old controls (*n* = 8), OFL—old rats exposed to FL (*n* = 9).

**Figure 2:**
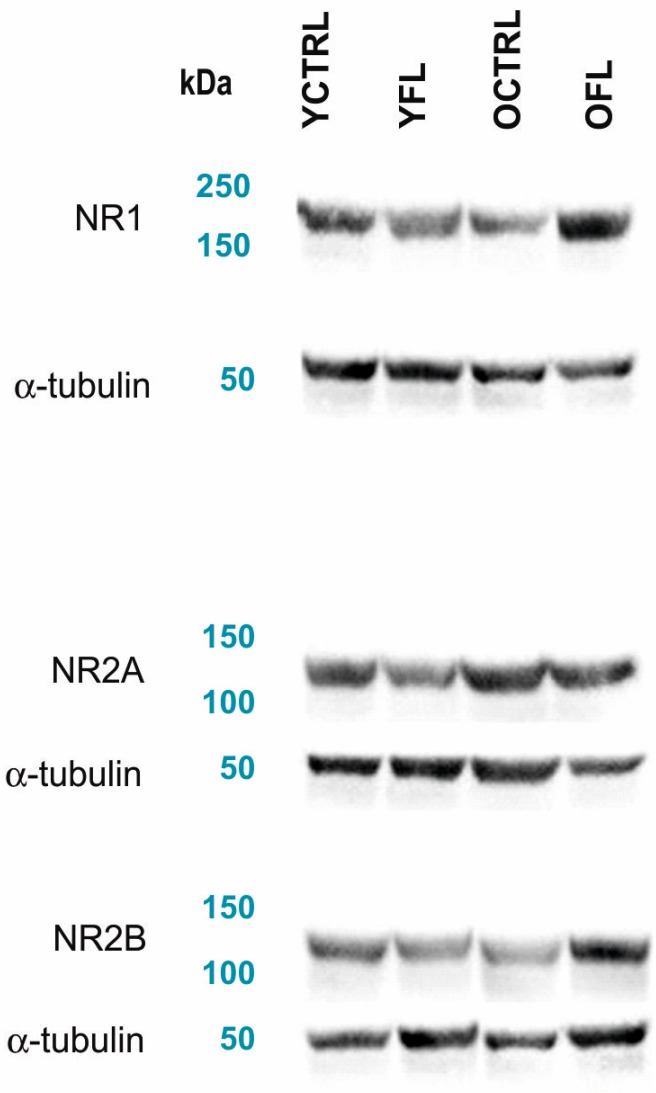
Representative images of Western blots from experiments on young adult and old rats exposed to FL (all data are presented in Figure 1). Representative Western blots showing the expression of NMDA receptor subunits, used for quantification of their changes in the frontal cortex. Anti-NMDA-NR1, anti-NMDA-NR2A and anti-NMDA-NR2B (all fromMerck) were used as primary antibodies. The loading control was incubated with an anti-α-tubulin antibody (Exbio). FL—forced locomotion, YCTRL—young adult control, YFL—young adult rat exposed to FL, OCTRL—old control, OFL—old rat exposed to FL.

**Figure 3:**
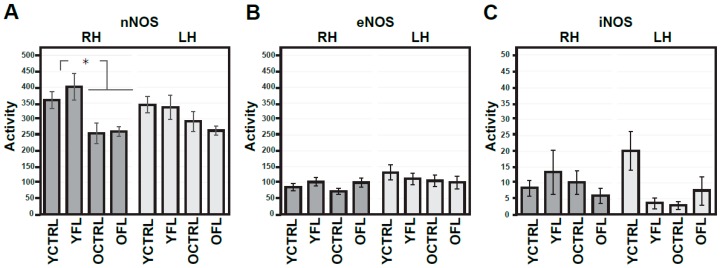
Activities of nitric oxide (NO) synthases in the right and left parietal cortex of young adult and old rats exposed in response to forced locomotion. Activities of various NOS isoforms (**A**–**C**) are expressed as nmoles/30 min/mg of proteins. Results are presented as means ± SEM. Statistical significance (Student’s *t*-test) was calculated with respect to young adult controls (* *p* < 0.05, ** *p* < 0.01). RH—right hemisphere, LH—left hemisphere, nNOS—neuronal NOS, eNOS—endothelial NOS, iNOS—inducible NOS, FL—forced locomotion, YCTRL—young adult controls (*n* = 8), YFL—young adult rats exposed to FL (*n* = 10), OCTRL—old controls (*n* = 8), OFL—old rats exposed to FL (n = 9).

NMDA receptors and downstream NOS signalling play an important role in activity-dependent modifications in synaptic connectivity and plasticity mechanisms. As a first step, we set out to investigate if changes in these molecular pathways can be detected in the cortex of Wistar rats exposed to FL, and whether these effects are age-dependent. Control experiments for the non-specific effects of the FL apparatus were also carried out, and used as a reference. Figure 1A–C presents a summary histogram of NR1, NR2A, and NR2B response to FL in the frontal cortex (Figure 2 shows representative images of Western blots from these experiments), while Figure 3A–C summarizes the response of downstream NOS isoforms in the R and L parietal cortex separately. Table 1 summarizes the indexes of laterality for particular NOS isoform activities.

Quantification of NR1, NR2A, and NR2B expressions in old compared to young rats showed significant age-dependent decreases of NR1 and NR2B (to 89.2% and 95.0%, respectively), while the expression levels of NR2A were age-insensitive. In the same series of experiments, young animals exposed to FL showed a significant decrease in expression of all NR1, NR2A, and NR2B subunits when compared to age-related controls (the drops to 95.7%, to 97.9% and finally to 97.9%). Surprisingly, in old age groups, exposure to FL caused a notable increase in NR1 and NR2B (to 110.2% and 106.1%, respectively) while NR2A expression remained unchanged (Figure 1A–C).

Similarly, we analysed the effects of FL on the activity of NOS isoforms, given that NO is one of the key downstream messengers of NMDA receptors. The results are summarized in Figure 3A–C. Measurements with ANOVA with repeated measures, one-way ANOVA, and t-test suggest more pronounced age-related decrease in nNOS activity on the R side (the drop to 71.1%). No age-related changes were observed in eNOS or iNOS activity. Moreover, we did not detect changes in the activity of any of these NOS isoforms related to FL. On the other hand, mild laterality in activity of all NOS isoforms, measured as lateralization index, were found in young adult rats (Table 1).

### 2.2. Changes in NMDA Receptors Subunits and NOS Activities Related to Acute SD (Experiment II)

**Figure 4:**
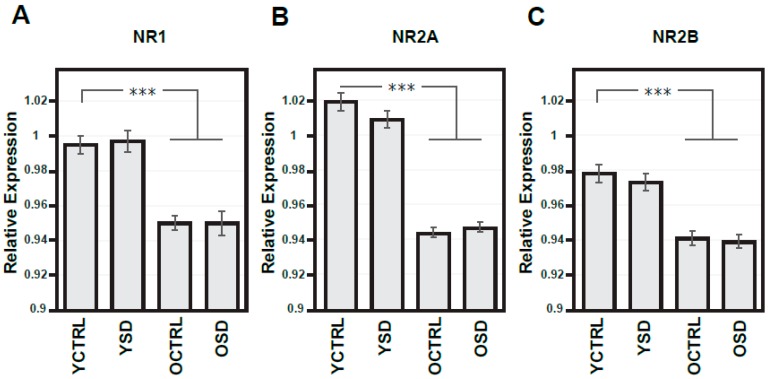
Expressions of NMDA receptor subunits in the frontal cortex from both hemispheres in young adult and old rats exposed to acute sleep deprivation. Optical density of samples respective NMDA subunit bands (**A**–**C**) related to that of α-tubulin; results are presented as means ± SEM. Statistical significance (Student’s *t*-test) was calculated with respect to young adult controls (*** *p* < 0.001). SD—sleep deprivation, YCTRL—young adult controls (*n* = 12), YSD—young adult rats exposed to acute SD (*n* = 12), OCTRL—old controls (*n* = 12), OSD—old rats exposed to acute SD (*n* = 12).

**Figure 5:**
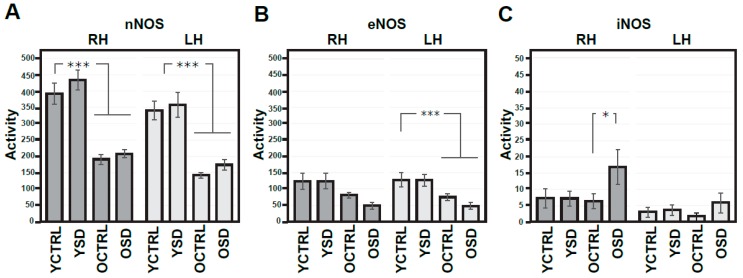
Activities of NO synthases in the right and left parietal cortex of young adult and old rats exposed to acute sleep deprivation. Activities of various NOS isoforms (**A**–**C**) expressed as nmoles/30 min/mg of proteins. Results are presented as means ± SEM. Statistical significance (Student’s *t*-test) was calculated with respect to young adult or old controls (* *p* < 0.05, *** *p* < 0.001). RH—right hemisphere, LH—left hemisphere, nNOS—neuronal NOS, eNOS—endothelial NOS, iNOS—inducible NOS, SD—sleep deprivation, YCTRL—young adult controls (*n* = 12), YSD—young adult rats exposed to acute SD (*n* = 12), OCTRL—old controls (*n* = 12), OSD—old rats exposed to acute SD (*n* = 12).

NOS signalling is known to be a key regulator of the sleep, while NMDA receptors-dependent mechanisms have been implicated in global regulation of the sleep–wake cycle and mechanisms of memory consolidation and brain aging. We set to investigate the impact of age and acute SD on NR1, NR2A, and NR2B expression in the frontal cortex (Figure 4A–C) and NOS isoforms activity in the R and L parietal cortex separately (Figure 5A–C). Table 1 presents indexes of laterality for NOS isoform activities. As can be readily seen, there was a considerable age-dependent reduction of the expression levels of all NMDA receptor subunits tested, with statistically significant differences in all three sets of data (the decrease to 95.5% in NR1, to 92.6% in NR2A, and finally to 96.2% in NR2B). No changes were observed in either young adult or old groups exposed to acute SD (Figure 4A–C).

In a similar way, assessments and cross-comparison of NOS isoforms in animals of different age groups with and without exposure to acute SD showed considerable age-dependent decrease but only in nNOS (the bilateral drops to 48.8% and 41.6%) and eNOS activity (the significant drop to 58.7% in the L side, that with borderline significance to 65.1% in the R side), with the activity levels of iNOS remaining stable across different age groups (Figure 5A–C). In young rats exposed to acute SD, no changes in the activity of NOS isoforms were found when compared to age-related controls. However, results of ANOVA with repeated measures and of t-test (but not of one-way ANOVA) supported the significant change in iNOS activity in the R side of old rats exposed to acute SD when compared to age-matched controls. Accordingly, lateralization index analysis revealed mild asymmetry in all NOS isoforms in young adult control rats (Table 1). Results of correlation analysis performed on data of experiment II did not support significant changes in young or old rats exposed to acute SD when compared to corresponding age-related controls.

### 2.3. Changes in NMDA Receptors Subunits and NOS Activities Related to Chronic SD (Experiment III)

**Figure 6:**
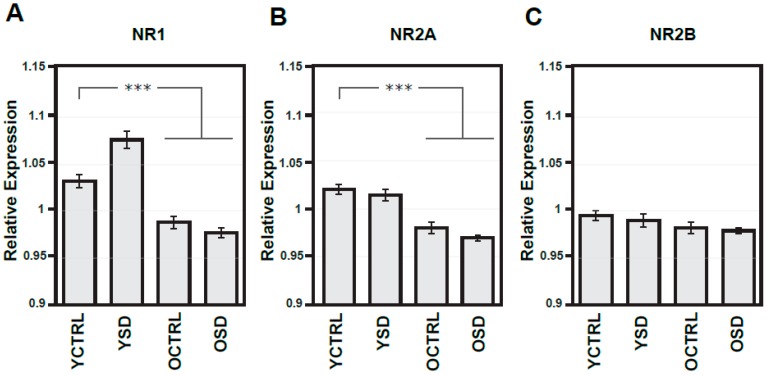
Expressions of NMDA receptor subunits in the frontal cortex from both hemispheres in young adult and old rats exposed to chronic sleep deprivation. Summary histograms illustrating the expressional changes in NR1 (**A**), NR2A (**B**) and NR2B (**C**) subunits. The optical density of samples of respective NMDA subunit bands was related to that of α-tubulin, results are presented as means ± SEM. Statistical significance (Student’s *t*-test) was calculated with respect to young adult controls (*** *p* < 0.001). SD—sleep deprivation, YCTRL—young adult controls (*n* = 12), YSD—young adult rats exposed to chronic SD (*n* = 12), OCTRL—old controls (*n* = 12), OSD—old rats exposed to chronic SD (*n* = 12).

**Figure 7:**
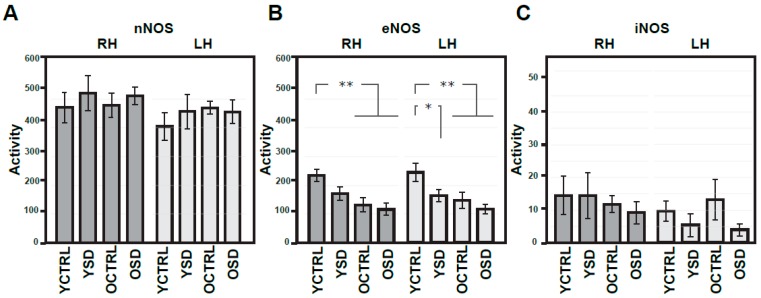
Comparison of activities of NO synthases in the right and left parietal cortex of young adult and old rats exposed to chronic sleep deprivation (experiment III). Summary histogram of activities of NOS isoforms (**A**–**C**) expressed as nmoles/30 min/mg of proteins. Results are presented as means ± SEM. Statistical significance (Student’s *t*-test) was calculated with respect to young adult controls (* *p* < 0.05, ** *p* < 0.01). RH—right hemisphere, LH—left hemisphere, nNOS—neuronal NOS, eNOS—endothelial NOS, iNOS—inducible NOS, SD—sleep deprivation, YCTRL—young adult controls (*n* = 12), YSD—young adult rats exposed to chronic SD (*n* = 12), OCTRL—old controls (*n* = 12), OSD—old rats exposed to chronic SD (*n* = 12).

Next, we investigated the impact of long-term SD on NR1, NR2A, and NR2B expression in the frontal cortex, and NOS isoforms activity in the R and L parietal cortices in two age groups. The results of these experiments are summarized in Figure 6A–C, Figure 7A–C, and in Table 1. With regard to NMDA receptor subunits, significant decline in NR1 and NR2A expressions (to 95.8% and 96.7%, respectively) were found in old compared to young adult controls. Mild decrease to 98.7% in NR2B reached only to borderline significance. Chronic SD-related changes in NMDA receptor subunits were not observed in young or old rats (Figure 6A–C).

Results of ANOVA with repeated measures, one-way ANOVA, and t-test supported age-dependent changes only in eNOS activity (bilateral drops to 55.7% and to 60.6%, respectively) in experiment III (Figure 6A–C). In young rats exposed to chronic SD, changes in eNOS activity (reduction to 66.7% in the L side and 72.8% in the R side) were found when compared to age-related controls. The lowest levels of eNOS activity were observed in old rats exposed to chronic SD. Nevertheless, the values of t-test did not reach statistical significance as compared to old controls. Moreover, a notable R/L laterality was observed in nNOS and iNOS activity but not in iNOS activity in young adult controls (Table 2). Correlation analysis performed on these experimental data did not reveal changes in young adult rats exposed to chronic SD as compared to age-related controls (In old rats exposed to chronic SD, however, two significant changes involving NR2A were observed: (i) the association of NR2A and NR2B subunits was reduced (old controls: CC = +0.978, *p* < 0.001, old exposed rats: CC = +0.674, *p* = 0.016, two-tailed test *p* = 0.002) and (ii) the association between NR2A and eNOS was enhanced (the R side—old controls: CC = −0.257, *p* = 0.419, old exposed rats: CC = +0.798, *p* = 0.002, two-tailed test *p* = 0.004; the very similar results with borderline significance were found also in the L side).

### 2.4. Age-Dependent Changes in NMDA Receptors Subunits and NOS Activities (Experiments I–III)

We analysed data of young adult and old controls from experiments I-III for age-dependent changes, with results summarized in Table 2. One-way ANOVA supported a significant drop in all subunit expressions (to 93.9% in NR1, to 96.3% in NR2A and finally to 96.9% in NR2B) and in some NOS isoforms activities (to 74.1% in the R side in nNOS, bilaterally to 62.3% and 63.4% in eNOS).

In young adult controls, results of correlation analysis revealed significant links between particular subunits of the NMDA receptor (the positive link between NR1 and NR2A (CC = +0.668, *p* < 0.001), between NR1 and NR2B (CC = +0.903, *p* < 0.001) or between NR2A and NR2B (CC = +0.762, *p* < 0.001)); between some subunits and NOS isoforms (the positive link between NR1 and iNOS in the L side (CC = +0.442, *p* = 0.011), or between NR2B and iNOS in the L side (CC = +0.483, *p* = 0.005)), and finally between particular NOS isoforms (the positive link between nNOS in the R and L side (CC = +0.637, *p* < 0.001); between eNOS in the R and L side (CC = +0.756, *p* < 0.001) and finally between nNOS in the L and eNOS in the R side (CC = +0.350, *p* = 0.049)). These positive links were not significantly altered in old control rats. On the other hand, correlation analysis revealed four significant changes in old compared to young rats associated with NR1/NR2B and nNOS in both sides: (i) the shift from the mild negative (CC = −0.182, *p* = 0.320) to the marked positive (CC = +0.528, *p* = 0.002) correlation between NR1 and nNOS in the R side (two-tailed test *p* = 0.003), (ii) the very similar change in the L side, i.e., the shift from the mild negative (CC = −0.092, *p* = 0.618) to the marked positive (CC = +0.626, *p* < 0.001) correlation between NR1 and nNOS in the L side, two-tailed test *p* = 0.002), (iii) the shift from the mild negative (CC = −0.108, *p* = 0.555) to the marked positive (CC = +0.451, *p* = 0.010) correlation between NR2B and nNOS in the R side (two-tailed test *p* = 0.024) and finally (iv) the very similar changes in the L side, i.e., the shift from the mild negative (CC = −0.054, *p* = 0.769) to the marked positive (CC = +0.589, *p* < 0.001) correlation between NR2B and nNOS in the L side (two-tailed test *p* = 0.005).

## 3. Discussion

In this study, we present several sets of experimental results demonstrating age- and SD-related changes in NMDA receptors subunits and downstream NO signalling in the cortex of male Wistar rats. While general alterations concerning these key molecular players are bilateral, some subtle molecular asymmetry was also evident. At this stage, the functional and homeostatic impact of described findings remains unknown. In light of the general impact of NMDA receptors and NOS enzymes on neuronal functions and synaptic plasticity mechanisms, it is, however, clear that described changes could contribute towards age-dependent deficit in synaptic plasticity and cognitive decline, with implications for pathobiology of AD and neurodegenerative process.

Prior to discussing aging or SD-related alterations, it is worth noting two interesting traits observed in young adult rats with reference to NMDA receptors and NOS activities: (i) moderate lateralization of nNOS and eNOS activity in the parietal cortex of young adult controls (Table 1) and (ii) mild seasonal variations in the expression levels and activity of NOS. Mild asymmetry of nNOS and eNOS isoforms agrees with our previous study in rats and humans [38,45]. Interestingly, the molecular asymmetry seems to be sensitive to seasonal changes, which typically is associated with changes in sex hormone levels [38,46]. Incidentally, functional asymmetry of nNOS and eNOS isoforms corresponds with published data (e.g., testosterone and other androgens are able to activate nNOS [47] and eNOS activity [48]). Seasonal fluctuations could possibly also contribute to the variability of some controls in our study, given that our experiments were performed in different time of the year on different animal cohorts. Our results also show an interesting association between changes in various NMDA subunits (NR1/NR2A, NR1/NR2B, and NR2A/NR2B) with some functional implications, in accordance with the literature [29]. A stronger coupling of NR1/NR2B (but not NR2A) and iNOS activity in the parietal cortex, for example, suggests that NR1/NR2B heteromers could be more tightly bound with iNOS at the postsynaptic side of glutamatergic synapses [49].

Analysis of NR1, NR2A, and NR2B subunits show a considerable difference in their sensitivity to age. NR1 decrease was evident in all three sets of experiments and is in agreement with previously published studies [27,28,29]. The age-dependent decrease was also found in NR2A or NR2B, although this effect was less pronounced. Assessment of the activity of NOS isoforms at matching time points revealed less convincing data for age-dependent changes. Thus, only in two of three experiments, alterations in nNOS or eNOS levels have been detected, while the level of iNOS remained stable. Analysis of data from all control rats revealed a decrease in nNOS and in eNOS activity (Table 2), which correlated with age-dependent modifications in NR1 and NR2B subunits. Taken as a whole, while there are consistent age-related changes in expression of NMDA receptor subunits and NOS signalling throughout the rat brain, our data do not fully support the HAROLD model, reporting higher sensitivity of the L hemisphere to processes of normal aging [38,46].

Similar to aging, NMDA receptor level and NOS signalling showed sensitivity to acute and chronic SD. In young adult rats exposed to acute 24 h SD, we could not reproduce increases in NMDA receptor expression observed by others, after 4 h SD, or reductions observed after 72 h SD [23,24,25]. In the older animal group, however, we have found a significant increase in iNOS activity in rats exposed to acute SD when compared to age-related controls, known to be induced by prolonged wakefulness. Data in the literature suggest that acute SD-evoked increase in NO via iNOS is an important homeostatic factor [39,40]. While it is remarkable that some of the molecular changes in NMDA receptor and NOS signalling show modest lateralization and agree with the results of previous studies in rats [38] and human [45], the neurophysiological significance and the role of these trends for normal brain functions and for pathobiology of disease remain unclear and require further research. As part of the heteromeric NMDA receptor complex, changes in the level of subunits imply their remodelling and functionality alterations, given that each type of NR2 subunit (e.g., NR2A and NR2B) plays a key role in overall receptor and channel function. The presence of the NR2A subunit, for example, shortens the channel open time, and affects receptor kinetics, with potential impact on long-term plasticity in working memory, reversal of learning, and sensory gating [49]. Therefore, age- and SD-related alterations would have implications for synaptic plasticity and biology of neurons. It seems that normal aging enhances negative effects of acute SD via iNOS signalling, and thus could evoke similar changes, as seen in AD [45]. Marked endothelial dysfunction evoked by chronic SD via decreased activity of neuroprotective eNOS, downstream to NMDA receptor activation, could also contribute to AD pathogenesis since this mechanism is affected by normal aging.

In this context, it is reassuring that in our control experiments using FL, some alterations of NMDA receptor subunit expression were also observed (Figure 1A–C, Figure 2), unlike SD experiments, implying a fundamental difference of neurobiological mechanisms of these two processes and their functional implications. Moreover, FL did not cause any changes in NOS activities, also in contrast to the data from SD experiments. To the best of our knowledge, the mechanisms of FL-related alterations in these key signalling molecules remain unexplored. It should be noted, however, that part of these changes could be due to stress response to the exercise, with increased circulation of corticosterone, known to influence the NMDA receptor subunit transcripts levels, and expression as receptors [50,51,52]. Differential mechanism of FL-related alterations from age and SD-dependent changes in NMDA subunits is also supported by the fact that FL did not mimic modifications related with aging and exposure to acute or chronic SD.

In summary, presented herein age and SD related modifications in NMDA receptors and NOS activity imply their sensitivity to aging, SD, and general well-being, with potential contribution of described processes to the cognitive decline in the course of normal aging and AD, promoting the neurodegenerative process. With stakes remaining high, future research is required to elucidate precise molecular players regulating these major signalling systems in aging and SD brains, which are of key relevance to basic neurobiology and translational neuroscience.

## 4. Materials and Methods

### 4.1. Animals

All experiments were performed on young adult (3–4 months old) and old (11–12 months old) male Wistar rats. Animals were housed in cages (2 young adult or old rats per cage) in a temperature-controlled room (21–22 °C), with a 12:12 h light/dark regime (lights on at 6:00 a.m.) and free access to food (ST-1 diet) and water. After finishing all exposures, rats were sacrificed by cervical dislocation, decapitated, and the brains were removed rapidly from the skulls. The cortices from both hemispheres were dissected on an ice-cold plate, divided into frontal and parietal parts, weighed (two frontal parts together, the parietal parts from the R and L side separately), wrapped up in aluminium, and frozen at −40 °C until assayed (for no more than 1 months). All manipulations were performed according to the Guidelines of the European Union Council (86/609/EU). The procedures for animal experimentation in this study were reviewed and approved (7 March 2013) by the Institutional Animal Care and Use Committee and were consistent with the Czech Government Requirements under the Policy of Human Care of Laboratory Animals (No. 246/1992) and with subsequent regulations from the Ministry of Agriculture of the Czech Republic.

### 4.2. Sleep Deprivation Experiments

All exposures were performed by means of Rat Forced Exercise Bed model 80805A*C apparatus (Campden Instruments) which is placed in the room with LD12:12 (lights: from 6 a.m. to 6 p.m., dark: from 6 p.m. to 6 a.m.). The aim was to model typical sleep disturbances observed in AD (especially fragmentation). Conditions of first two sets of experiments were similar to those in literature [53] and were as follows: experiment I—exposure of rats to forced locomotion (FL), the control experiment for the non-specific side effects of the activity wheel: test time: 7 m/min for 3 h, from 8.00 a.m. to 11.00 a.m. (relatively fast and uninterrupted rotation for 3 h); experiment II—exposure of rats to acute SD: totally 24 h, test time: 4 m/min for 4 s, rest time: 60 s, from 8.00 a.m. to 8.00 a.m. (slow and interrupted rotation for 24 h); experiment III—exposure of rats to chronic SD: in total 12 days, exposure for 20 h a day (test time: 4 m/min for 4 s, rest time: 60 sec, from 11.00 a.m. through the night to 8.00 a.m.), rest for 4 h a day (animals rested without turning from 8.00 a.m. to 11.00 a.m.) (slow and interrupted rotation for 20 h a day, totally 12 days). Experiment I was performed on 18 young (8 controls and 10 exposed) and 17 old (8 controls and 9 exposed) rats, experiment II was performed on 24 young (12 controls and 12 exposed) and 24 old (12 controls and 12 exposed) rats, and finally experiment III was performed on 24 young (12 controls and 12 exposed) and 24 old (12 controls and 12 exposed) male Wistar rats.

### 4.3. Analytical Biochemistry Experiments

Expression of the NMDA receptors subunits NR1, NR2A, and NR2B in the frontal cortex were quantified using Western blotting. In brief, all samples were suspended in a loading buffer (63 mM Tris; 10% glycerol; 2% sodium dodecyl sulphate, 5% 2-mercaptoethanol and 0.01% bromophenol blue). The re-suspended material was used for the electrophoresis in the 7.5% polyacrylamide gel (Bio-Rad, Hercules, CA, USA) followed by electro-blotting in the criterion blotter (Bio-Rad, Hercules, CA, USA). Nonspecific binding was blocked with 3% non-fat milk (Merck, Kenilworth, NJ, USA) and dissolved in 0.1% Tween in phosphate-buffered saline (PBS buffer)/Tris-buffered saline (TBS buffer). Blots were incubated with anti-NMDA-NR1 (Merck, Kenilworth, NJ, USA, 1:1000 in 5% bovine serum albumin in 0.1% Tween in TBS buffer, overnight incubation), anti-NMDA-NR2A (Merck, Kenilworth, NJ, USA, 1:500 in 0.1% Tween in TBS buffer, 2 h incubation at room temperature) and with anti-NMDA-NR2B (Merck, Kenilworth, NJ, USA, 0.1% Tween in TBS buffer, 2 h incubation at room temperature) as primary antibodies and the loading control with an anti-α-tubulin antibody (Exbio, Prague, Czech Republic, 1:1000 in 0.1% Tween in PBS buffer, 1 h incubation at room temperature). After, all blots were washed in PBS buffer/TBS buffer and incubated for 1 h with a horseradish peroxidase-conjugated secondary antibody (Dako, Santa Clara, CA, USA, 1:3000, for α-tubulin—dilution of 1:5000 was used). Detections were performed with a chemiluminescent substrate (ThermoFisher Scientific, Walthman, MA, USA) and evaluated by the Gel Doc Analysis system (Bio-Rad, Hercules, CA, USA), in accordance with our previous studies [29,36].

The activities of nNOS, eNOS, and iNOS were estimated in the R and L parietal cortex separately using chemicals (except for radiolabelled arginine) from Merck, Kenilworth, NJ, USA. In brief, the parietal cortices were homogenized (1:10) in homogenization buffer (1 mM EGTA, 1 mM dithiothreitol, 20 mM HEPES, 0.32 M sucrose, 14.6 µM pepstatin and 21 µM leupeptin, pH = 7.4) and the resulting homogenates centrifuged at 1200× *g* for 10 min at 4 °C. Supernatants were added to the reaction buffer (homogenization buffer containing also 200 µM β-nicotinamide adenine dinucleotide phosphate, 50 µM tetrahydrobiopterin and 4.6 µM [14C]arginine (ARC, St. Louis, MO, USA,)) and incubated for 30 min at 37 °C. Some samples also contained 1 µM CaCl_2_ (nNOS and eNOS) and specific inhibitors (1 mM spermidine for nNOS, 190 µM Nω-nitro-l-arginine methyl ester for nNOS/eNOS and 1 mM aminoguanidine for iNOS). Final protein concentrations determined by the Bradford method equalled 0.5 mg/mL in all incubation mixtures. The reaction was terminated by adding the stop buffer (30 mM HEPES, 3 mM EDTA, pH = 5.5) and by rapid cooling. DOWEX 50WX8-200 was used to separate citrulline from arginine, in accordance with our previous studies [29,38,45].

### 4.4. Statistical Analysis

The BMDP statistical software was used for data analysis and comparison. ANOVA with repeated measures (program 2V), one-way ANOVA, and Student’s *t*-test (program 7D) and correlation analysis (program 6D) were applied for comparison of various data samples. Differences between two independent values of correlation coefficients (CCs) were calculated by VassarStats website using the Fisher r-to-z transformation (two-tailed test). In addition, the index of laterality (L − R)/(L + R) was calculated to estimate differences between the R and L side. The index is limited to zero when all values are not lateralized or when the numbers of markedly R/L (dominance of the R side) and L/R (dominance of the L side) lateralized animals are approximately equal. Mild laterality was defined in this study by the index of laterality > /± 0.050/, marked laterality by that > /± 0.100/. Data in the tables are presented as the means ± S.E.M.

## Figures and Tables

**Table 1:** Indexes of laterality of activities of NOS isoforms in the R and L parietal cortex.

Groups	Index of Laterality for nNOS	Index of Laterality for eNOS	Index of Laterality for iNOS
**Experiment I**			
young adult controls (*n* = 8)	−0.034 ± 0.033	+0.188 ± 0.077	+0.196 ± 0.235
young adult exposed to FL (*n* = 10)	−0.072 ± 0.082	+0.020 ± 0.094	−0.128 ± 0.232
old controls (*n* = 8)	+0.067 ± 0.065	+0.156 ± 0.064	−0.239 ± 0.226
old exposed to FL (*n* = 9)	−0.007 ± 0.040	−0.029 ± 0.102	+0.084 ± 0.306
ANOVA:	*p* = 0.448	*p* = 0.266	*p* = 0.637
**Experiment II**			
young adult controls (*n* = 12)	−0.064 ± 0.033	+0.035 ± 0.088	−0.163 ± 0.270
young adult exposed to acute SD (*n* = 12)	−0.113 ± 0.055	+0.093 ± 0.098	−0.282 ± 0.249
old controls (*n* = 12)	−0.136 ± 0.058	−0.054 ± 0.061	−0.385 ± 0.137
old exposed to acute SD (*n* = 12)	−0.077 ± 0.092	−0.007 ± 0.161	−0.396 ± 0.184
ANOVA:	*p* = 0.846	*p* = 0.800	*p* = 0.860
**Experiment III**			
young adult controls (*n* = 12)	−0.113 ± 0.046	+0.005 ± 0.052	−0.180 ± 0.229
young adult exposed to chronic SD (*n* = 12)	−0.128 ± 0.038	−0.011 ± 0.072	−0.263 ± 0.229
old controls (*n* = 12)	+0.007 ± 0.034	+0.044 ± 0.074	−0.131 ± 0.179
old exposed to chronic SD (*n* = 12)	−0.073 ± 0.046	+0.057 ± 0.071	−0.137 ± 0.231
ANOVA:	*p* = 0.109	*p* = 0.879	*p* = 0.971

Results are presented as means ± SEM. Indexes of laterality were calculated from data of Figure 2, Figure 4 and Figure 6. Experiment I: experiments on young adult animals were performed in January, those on old animals in November. Experiment II: experiments on young adult animals were performed in April and May, those on old animals in December. Experiment III: experiments on young adult animals were performed in June, those on old animals in February.

**Table 2:** Age-dependent changes in NMDA receptor—NO system.

Components of NMDA—NO Pathway	Young Adult Controls (*n* = 32)	Old Controls (*n* = 32)	ANOVA: p
NR1 expression	1.034 ± 0.008	0.971 ± 0.004	<0.001 ***
NR2A expression	1.026 ± 0.004	0.988 ± 0.009	<0.001 ***
NR2B expression	1.000 ± 0.005	0.969 ± 0.005	<0.001 ***
nNOS activity in the R side	423.3 ± 26.2	313.6 ± 29.3	=0.007 **
nNOS activity in the L side	361.6 ± 22.0	294.9 ± 28.8	=0.071
eNOS activity in the R side	155.9 ± 17.0	97.2 ± 11.1	=0.005 **
eNOS activity in the L side	172.8 ± 18.1	109.5 ± 13.4	=0.007 **
iNOS activity in the R side	9.2 ± 2.2	8.5 ± 1.5	=0.800
iNOS activity in the L side	9.5 ± 2.2	6.0 ± 2.3	=0.272

Results are presented as means ± SEM. Differences were calculated from data of young adult and old controls presented in Figure 1, Figure 2, Figure 3, Figure 4, Figure 5 and Figure 6. Statistical significance (ANOVA) was calculated with respect to young adult controls (* *p* < 0.050, ** *p* < 0.010, *** *p* < 0.001). Results of ANOVA with repeated measures:
nNOS: aging—F(1,62) = 6.23, *p* = 0.015, laterality—F(1,62) = 8.87, *p* = 0.004, interaction—F(1,62) = 2.54, *p* = 0.116. eNOS: aging—F(1,62) = 9.23, *p* = 0.004, laterality—F(1,62) = 3.79, *p* = 0.056, interaction—F(1,62) = 0.09, *p* = 0.761. iNOS: aging—F(1,62) = 0.99, *p* = 0.325, laterality—F(1,62) = 0.30, *p* = 0.589, interaction—F(1,62) = 0.51, *p* = 0.477.

## Abbreviations

SD	sleep deprivation
AD	Alzheimer’s disease
NMDA	N-methyl-D-aspartate
NO	nitric oxide
REM	rapid eye movement
FL	forced locomotion
nNOS	neural nitric oxide synthase
eNOS	endothelial nitric oxide synthase
iNOS	inducible nitric oxide synthase
Aβ	amyloid β peptide
PBS buffer	phosphate-buffered saline
TBS buffer	Tris-buffered saline
R	right
L	left
